# Foreign Body in the Air Passage for Nine Months—Expulsion—Cure

**Published:** 1842-06

**Authors:** 


*Foreign Body in the Air Passage for Nine Months—Expulsion—Cure.*—M. Malshieurat-Lagemard communicated to the Academy of Medicine of Paris an interesting case of this character. The subject of it was a woman 58 years of age, vigorous constitution, who when first seen by M. M. had been laboring under a cough for four months. At the commencement of the attack the fits of coughing were violent and accompanied with a sense of suffocation. When she consulted M. M. "the cough recurred every eight or ten days, lasted for one or two minutes, and was unaccompanied with fever or expectoration. The patient could assign no cause for the attack, nor was any thing learned by auscultating the thorax. The symptoms continued without aggravation, or mitigation, and finally, the patient expectorated along with some mucus, a rough, yellowish calculus, about the size of a filbert nut. On sawing the calculus, it was found to consist of calcareous matter, encrusted on a cherry stone. The patient then recollected that the first attack of cough occurred when she was eating cherries, but if she experienced any sensation of suffocation at the time, it was so slight as to have quite escaped her memory. She remained perfectly well after expulsion of the calculus."

In a case related in the *Ephem. Nat. Cur.* a cherry-stone remained in the air passages for a year, and was then expelled by coughing, encrusted by tartar.—*Am. Journn. Med. Sci.*

